# Application of the Free Tangent Law in Quantification of Household Satisfaction from Durable Consumer Goods

**DOI:** 10.3390/e23091109

**Published:** 2021-08-26

**Authors:** Wiktor Ejsmont, Marek Biernacki

**Affiliations:** 1 Mathematical Institute, University of Wroclaw, pl. Grunwaldzki 2/4, 50-384 Wroclaw, Poland; 2 Department of Mathematics and Cybernetics, Wroclaw University of Economics and Business, Komandorskast. 118/120, 53-345 Wrocław, Poland; marek.biernacki@ue.wroc.pl

**Keywords:** the free tangent law, subjective wellbeing, durable goods

## Abstract

This paper presents a postulate for a new approach in the measurement of households’ satisfaction from durable consumer goods, based on a modified inflation expectation measurement method used in survey research. The authors examine the application of a three-step qualitative evaluation, followed by the quantification of responses using a modified Carlson and Parkin method adopted in the context of the free tangent law.

## 1. Introduction

This paper references the problem of quality of life evaluations as measures of social development defined in terms of growth in social welfare. The economic sciences typically employ a purely economic interpretation of social welfare, expressed in the utility of disposable income to satisfy the provision of goods and services [1]. For several decades now, quality of life improvements have been emphasized as one of the main objectives of progress in developed regions. For the EU area, the pertinent declarations were expressed in the Maastricht Treaty (1992) and the Treaty of Lisbon (2007).

Practical evaluations of such notions as quality of life or happiness (satisfaction) should not be based solely on their economic properties or effects. As evidenced in a study by [2], with the doubling of the US *per capita* GDP recorded over the last three decades of the 20th century, the average level of satisfaction in the period under examination seemed to follow a more or less constant trend. Thus, by constraining the measure of social welfare to purely pecuniary and income-based indices (such as the per capita GDP), we face the risk of false conclusions. In practice, many different measures are commonly employed for the evaluation of social welfare, and various life aspects are taken into consideration. For instance, the living conditions index includes such key drivers as housing conditions, dietary habits (calorie intake per person), health care and physical wellbeing, involvement in sports and cultural activities, ecology, mobility, and work respite (duration and preferences). The quality of life index (QOL) supplements these with measures of security and safety (murder and rape rates), quality of family and social relations (divorce rates), working conditions, migration, etc., but also by a number of economic measures, such as per capita GDP, unemployment rates, differences in income distribution, and poverty. The human development index (HDI) is designed to reflect the level of income, education, and health in the general population. Biernacki and [3] provide evidence to confirm the observation that the dynamics of quality of life development in the years 2006–2015, measured by a modified HDI index for the EU area, was significantly more pronounced in the segment of developing economies (compared to that in mature and decidedly more affluent economies).

There is extensive interest in the use of welfare in social science, such as economics. The literature review on this topic in economics was elaborated by [4]. The covariate responses to subjective welfare questions have been discussed, for instance, by [5,6]. A commonly used method in data analysis is the regression of the survey responses on individual and household responses. It was observed that interpersonal comparisons of welfare using subjective data have an impact on measurements.

This paper presents arguments for the application of a new method of measurement, considering not only the living conditions but—most of all—satisfaction of respondents from durable consumer goods held in their possession (household effects). The postulated approach serves to reconcile the inconsistencies (lack of correlation), evident in [2], between the value of household effects held and the satisfaction from such effects. Ferrans [7] (see also [8]) used measurements of quality of life and the satisfaction gotten from different areas of life. The quality of life was defined as “a person’s sense of wellbeing that stems from satisfaction or dissatisfaction with the areas of life that are important to him/her”.

Durable goods play a decisive role in the satisfaction of household needs and requirements. Many durable consumer goods, previously classified as luxury items, have become universally accessible and widely used on a daily basis by more households. These goods include a washing machine, a vacuum cleaner, and an icebox. Place of residence, neighbourhood and house/flat ownership remain the most important elements in the structure of material possession of households. Other elements of major significance include the ownership of a second lodging and ownership of personnel vehicles.

The main objective of this paper is the effective evaluation of satisfaction from material affluence among households. It is a well-established idea that people assess their welfare relative to some “comparison group” such as neighbours or co-workers (e.g., [9,10]. This paper presents a new approach to the measurement of living conditions, observably different from the pool of the available solutions, c.f.: [11,12]. The most notable difference is the elimination of the pecuniary value of household effects; instead, the method examines their role in the satisfaction of household needs and requirements. Another important distinction of the postulated approach is the application of the free tangent law [9] for the analytical evaluations of responses.

Based on the responses from questionnaire surveys, households were divided into three groups. The first group was represented by low-income households reporting problems in procurement of some of their basic material needs, either due to the inadequacy of resources or low creditworthiness. This group also included those households which, while reporting ownership of durable goods, regarded them well below their expectations. The second group was populated by households reporting their indifference to material needs or actively rejecting decisions to purchase certain goods despite having access to adequate resources. The third group represented households with access to durable goods under study and attesting to their satisfaction from them.

For the postulated approach to quality of life measurements, it is assumed that simple questions and limitation of response variants seem to produce a better reflection of the genuine feelings and concerns of the respondent households than the image offered by more complex queries with rich sets of available responses; this is similar to the effects observed in association with inflation expectations. The quantification method is used for the evaluation of survey responses of a purely qualitative nature; this is intended to ensure comparability of data obtained from each of the studied groups. Thus, models employed for the evaluation of inflation expectations, after suitable modifications, proved their usefulness when applied to the quality of life assessment in households.

## 2. The Free Tangent Law

The free tangent law is a notion postulated in [9]. Before we proceed with a detailed presentation of this type of distribution, it may be useful to recall some of the basic concepts of the free probability theory, developed in the early 1980s in [13].

A tracial noncommutative probability space is a pair $\left(A,\tau \right)$ where A is a unital algebra, and τ:A→C is a normal, faithful, tracial state. The elements X∈A are called (noncommutative) random variables; all random variables are assumed to be self-adjoint $X\in A_{sa}$. Given a noncommutative random variable $X\in A_{sa}$, the spectral theorem provides a unique probability measure $\mu_{X}$ on ℝ, which encodes the distribution of X in the state τ, i.e., $\tau \left(f,\left(X\right)\right)=\int_{R}f\left(\lambda \right)\;{d\mu }_{X}\left(\lambda \right)$, for any bounded Borel function f on ℝ.

A family of subalgebras ${\left(A_{i}\right)}_{i\in I}$ of A is called *free* if $\tau \left(X_{1}\ldots X_{n}\right)=0$ whenever $\tau \left(X_{j}\right)=0$ for all j=1,…,n and $X_{j}\in A_{i\left(j\right)}$ for some indices $i\left(1\right)\neq i\left(2\right)\neq \cdots \neq i\left(n\right)$. Random variables $X_{1},\ldots ,X_{n}$ are freely independent (free) if the subalgebras they generate are free.

The analytic approach to free probability is based on the Cauchy transform $G_{\mu }\left(z\right)=\int_{R}\frac{1}{z-y}\;d\mu \left(\lambda \right)$ of a probability measure μ. Moreover, the Cauchy transform has an inverse value in theneighbourhood of infinity, which has the form $G_{\mu }^{-1}\left(z\right)=\frac{1}{z}+R_{\mu }\left(z\right),$ where $R_{\mu }\left(z\right)$ is analytic in a neighbourhood of zero, and is called an R-transform.

In [9], we postulate the examination of asymptotics of quadratic forms in free random variables. This approach introduces a distribution of free tangents as limits of free commutators.

**Theorem** **1.** **(** *Ejsmont, Lehner [9]* **).**

Let $X_{1},X_{2},\ldots ,X_{n}$ be free identically distributed random variableswith variance 1, then:

(1) $$Q_{n}=\frac{1}{n}\sum_{\underset{k<j}{k,j=1}}^{n}i\left(X_{k}X_{j}-X_{j}X_{k}\right)\overset{d}{\to }Y,$$

where $R_{Y}\left(z\right)=\tan\left(z\right)$. We call the limit law $\mu_{Y}$, the standard free tangent law.

**Figure 1:**
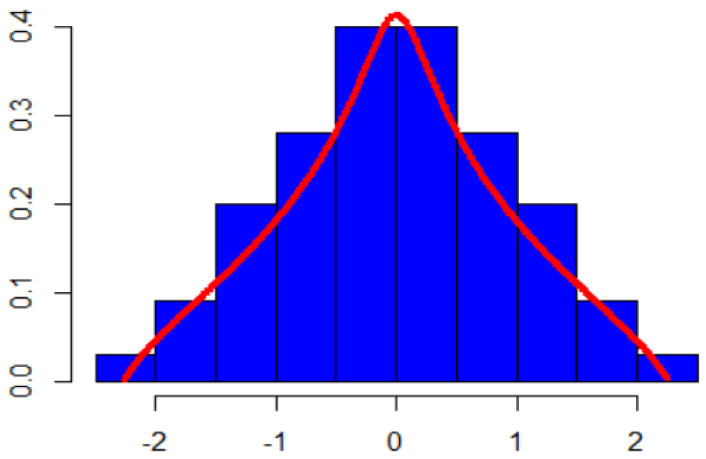
Histogram of survey results and the normalized distribution of tangents. Source: own research.

The free tangent law, with mean μ and variance, $\sigma^{2}$ is denoted by $F(\mu ,\sigma^{2})$, i.e., it has distribution μ+Yσ, where Y is the standard free tangent law. The cited work provides evidence to confirm the thesis that while the distribution density function cannot be formed explicitly, its shape may be deduced from numerical calculations (see Figure 1). The support (spectrum) for this type of distribution is defined as $\left(-\rho ,\;\rho \right)$, where ρ≃2.26. Proof of Theorem 1 was founded on the phenomenon of free commutator cancelling, presented in [14]. This type of distribution was also used as a limit for certain random matrices in [15], c.f. Section 7.2 with α = $\frac{\pi }{2}$.

Over the course of pilot studies, it was revealed that the free tangent distribution might effectively be employed as a measure of the material affluence of households. A group of 156 respondents were asked to describe the material situation of their households on a scale from 0 (very bad) to 1 (very good). The responses were then rescaled to fit the effective spectrum of the free tangent distribution and plotted on a histogram and a tangent distribution density function (red curve). In effect, the tangent distribution proved its usefulness in the evaluation of survey results (Figure 1) and served as direct motivation for the utilization of this type of distribution in qualitative studies of household satisfaction from durable goods.

The above accordance may be explained by the assumption that opinions of respondents with regard to material possessions are most probably formed in relation to the material situation of their neighbours. In effect, their responses may be seen as dependent variables since their expressed level of satisfaction was related to the welfare of others (acquaintances, friends, neighbours). It may then be assumed that the need for possession of certain types of goods is relatable to the level of conviction that the material welfare of a household is defined by the material position of other members of the respondent’s immediate social environment. Let us note that the distribution of tangents defines the limits of non-commutative random variables. By assuming that respondents, in their expressions of welfare preferences, are influenced by certain random factors of non-commutative character, the averaging results shall roughly correspond with the tangent distribution.

## 3. Analysis of Inflation Expectations

Inflation expectations are one of the factors of potential impact upon social behaviours related to consumption and savings, and thus, upon price fluctuation processes in economies. The knowledge of inflation expectations displayed by various groups of market participants is invaluable. For instance, it may be employed as a measure of bank credibility as an expression of public conviction and capabilities to fulfil objectives. One of the methods used in the provision of such data is the quantitative questionnaire survey study. Survey queries of quantitative character are usually formed as follows: *Judging by the current situation, what are your expectations of change in the general price level (in %) within the next 12 months?*

Data provided by qualitative surveys (or queries), on the other hand, are designed to reflect consumer attitudes and opinions related to their perception of future inflation process dynamics. As such, they are not meant to supply any direct or explicit measure of the expected level of inflation in the studied group. In order to obtain numerical values for the expected inflation trend dynamics without resorting to quantitative queries, we need to apply effective methods for the quantification of expectations expressed in a qualitative form. Two basic methodological approaches to the task at hand are readily available: the probabilistic method—see [16,17].

The probabilistic methods of quantification adopted in forecasts of inflation expectations are based on the assumption that the expected price level change in a population follows a certain distribution. Parameters of such distribution may be derived from information provided in qualitative responses. The earliest probabilistic models for the determination of inflation expectations from qualitative surveys were introduced in the second half of the 20th century in [17] More than two decades later [16] presented a substantially different procedure, although based on the use of the key components of [17] approach. Both postulates were designed to quantify 3-variant responses to qualitative queries of the expected changes in the price level.

The growing popularity of direct inflation-targeting strategies in the 1990s seemed to revitalize the design of probabilistic methods of quantification in analyses of inflation expectations. New procedures were developed to extend the informative qualities of responses, with respondents declaring not only the direction but also the intensity of the expected changes in the price level. The structure of the query adopted in the evaluation of individual inflation expectations, which originally provided three answer variants, has recently been expanded to offer a wider selection of choices. Examples of studies using this approach can be found in [18,19,20,21].

## 4. Model for the Measurement of Household Satisfaction from Durable Consumer Goods

This section outlines our postulated approach, based on a modified theory of inflation expectations as applied to the evaluation of satisfaction from material possessions among households. For the purpose of this method, it was assumed that the distribution of expectations $F(p_{n}^{e},\sigma_{n}^{2})$ follows the free tangent law.

The evaluation of household material welfare was formed from survey responses, representing opinions collected from *m* respondents over a set of *N* queries targeting various categories of durable consumer goods used in households. The study was designed to offer an effective measure of the quality of life reported by various populations of households, based on qualitative responses to questions related to their satisfaction with each category of goods.

For each *n*-th category of goods, the following general structure of query (and variants of answers offered) was adopted:

Do you use/have access to *n* in your household? If so, does it fulfil your expectations?

(a). I don’t (due to financial constraints), or I do, but it is well below my expectations,
(b). No, I don’t have a need for that,
(c). Yes, and it fulfils my expectations.

The theory of inflation expectation takes account of an important parameter termed the reference level $p_{n}$. For examination of inflation expectations, this represents the real inflation rate. For the purpose of this study, the value of this parameter was established from results presented in the last column of Table 1, reflecting the prevalence of the *n*-th category of goods, namely: $p_{n}=I+II-III$, with Roman numerals representing the respective columns of Table 1. Columns I and II store reports of satisfaction from *n* and declarations of disinterest in *n*; their summed value less the number of those who confirm their need but cannot afford the item, forms our reference level.

For each n-th category query, every *i*-th respondent formulates their own subjective distribution of the expected ‘quality’ of the *n*-th category elements in their possession with $p_{n}^{e}$ and variance $\sigma_{n}^{2}$. In general, it may be observed that this distribution is different for each respondent and may also vary in relation to the form of the query. For our purpose, it was assumed that preferences within the entire respondent population follow the free tangent law $F(p_{n}^{e},\sigma_{n}^{2})$.This distribution may thus be interpreted as a measure of a household’s satisfaction from material possessions.

In addition, it was assumed that the threshold of response functions −ε, ε are symmetric for each respondent, equal and query-independent, with ε, representing a certain positive-value constant under assessment. In the inflation expectation theory, the value range (−ε,ε) is referred to as the sensitivity range (e.g., [23]).

**Figure 2:**
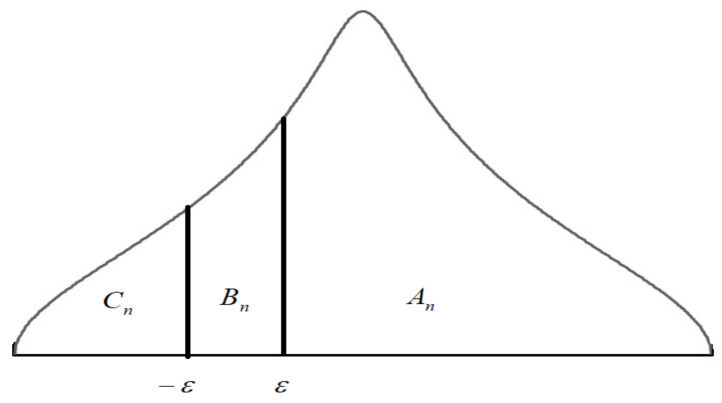
Graphical presentation of the assumptions of the Carlson–Parkin method. Source: own research based on [24].

The graphical representation (Figure 2) of the assumptions of the Carlson–Parkin method in the postulated model can be expressed by the following algebraic formulas:

(2) $$A_{n}=P\left(x_{i,n}^{}>\varepsilon \right)\;=1-G\left(\varepsilon \right),$$

(3) $$B_{n}=P\left(-\varepsilon <x_{i,n}^{}<\varepsilon \right)\;=G\left(\varepsilon \right)-G\left(-\varepsilon \right),$$

(4) $$C_{n}=P\left(x_{i,n}^{}<-\varepsilon \right)\;=G\left(-\varepsilon \right).$$

with G(⋅) representing a distribution function of the free tangent distribution.

Standardization of the free tangent distribution $F(p_{n}^{e},\sigma_{n}^{2}),$ using the formula:

(5) $$G(x)=\tilde{G}\left(\frac{x-p_{n}^{e}}{\sigma_{n}}\right),$$

yields the following for Formulas (2)–(4):

(6) $$1-A_{n}=\tilde{G}\left(\frac{\varepsilon -p_{n}^{e}}{\sigma_{n}}\right),$$

(7) $$B_{n}=\tilde{G}\left(\frac{\varepsilon -p_{n}^{e}}{\sigma_{n}}\right)-\;\tilde{G}\left(\frac{-\varepsilon -p_{n}^{e}}{\sigma_{n}}\right),$$

(8) $$C_{n}=\tilde{G}\left(\frac{-\varepsilon -p_{n}^{e}}{\sigma_{n}}\right).$$

Only two of the above, namely (6)–(8), are independent, i.e., one is a resultant of the remaining two. By limiting our examination to Formulas (6) and (7), with the additional assumptions:

(9) $$a_{n}=\frac{\varepsilon -p_{n}^{e}}{\sigma_{n}},$$

(10) $$b_{n}=\frac{-\varepsilon -p_{n}^{e}}{\sigma_{n}},$$

we obtain the following, after a series of simple operations:

(11) $$a_{n}={\tilde{G}}^{-1}(1-A_{n}),$$

(12) $$b_{n}={\tilde{G}}^{-1}\left(1-A_{n}-B_{n}\right)={\tilde{G}}^{-1}(C_{n}),$$

where ${\tilde{G}}^{-1}(x)$ is a function opposite to the distribution function of the standardized free tangent distribution $\tilde{G}(x).$

Further modifications of Formulas (8) and (9) accounting for $p_{n}^{e}$ and $\sigma_{n}$ and described by the value range of $a_{n},b_{n}$, yields a set of formulas that may be used to determine the expected value $p_{n}^{e}$ and the standard deviation $\sigma_{n}$ of the free tangent distribution $F(p_{n}^{e},\sigma_{n}^{2})$ describing the expected value of material possessions:

(13) $$p_{n}^{e}=\frac{-\varepsilon (a_{n}+b_{n})}{a_{n}-b_{n}},$$

(14) $$\sigma_{n}=\varepsilon \frac{2}{a_{n}-b_{n}}.$$

Estimations of the greatest credibility for parameters $A_{n},B_{n},C_{n}$ are derived from numerical data obtained from questionnaire surveys, i.e., the share of respondents subscribing to each variant having access to n-th category of goods and being satisfied by their qualities $\left({\hat{A}}_{n}\right)$; not having access and declaring no such need $\left({\hat{B}}_{n}\right)$; and not having access due to financial constraints or having access, but finding them below expectations $\left({\hat{C}}_{n}\right)$, with ${\hat{A}}_{n}+{\hat{B}}_{n}+{\hat{C}}_{n}=1$. Estimators ${\hat{A}}_{n},{\hat{B}}_{n},{\hat{C}}_{n}$ are only considered within each identified group of respondents. Thus, the estimators of the expected value $p_{n}^{e}$ and the standard deviation $\sigma_{n}$ may be expressed as follows:

(15) $${\overset{⌢}{p}}_{n}^{e}=\frac{-\varepsilon ({\hat{a}}_{n}+{\hat{b}}_{n})}{{\hat{a}}_{n}-{\overset{⌢}{b}}_{n}},$$

(16) $${\hat{\sigma }}_{n}=\varepsilon \frac{2}{{\hat{a}}_{n}-{\hat{b}}_{n}},$$

where:

(17) $${\hat{a}}_{n}={\tilde{G}}^{-1}(1-{\hat{A}}_{n}),$$

(18) $${\hat{b}}_{n}={\tilde{G}}^{-1}({\hat{C}}_{n}).$$

The above quantification procedure yields a function of the structure of responses to survey queries against the ε parameter that determines the spread of the sensitivity range. In the original model, the answer threshold ε, serving as a scaling parameter, constituted an exogenous variable, introducing the condition of a discretionary attribution value. A number of intuitive limitations were formulated and expected of the phenomenon of inflation expectations, and the sensitivity range was modified to ensure that the estimated values were placed within the pre-established limits. One of the more potent examples of such limitations is the introduction of assumed unbiasedness in inflation expectations to supplement the key assumptions of the Carlson-Parkin method. Per analogy to the inflation expectation methods, it was then assumed that the welfare expectations fulfil the criterion of:

(19) $$\sum_{n=1}^{N}p_{n}^{e}=\sum_{n=1}^{N}p_{n},$$

It must be noted that, by making the assumption(19), we face the risk of errors made by respondents in the formulation of their responses, but in general, they offset to zero. This is justified due to the postulated correctness and objectivity of the entire evaluation process. Thus, one of the potential consequences is the following estimation of the parameter ε:

(20) $$\overset{⌢}{\varepsilon }=\frac{\sum_{n=1}^{N}p_{n}}{\sum_{n=1}^{N}\frac{{\hat{a}}_{n}+{\hat{b}}_{n}}{{\hat{b}}_{n}-{\hat{a}}_{n}}},$$

(this equation was construed as sums of values from n=1 to n=N of Equation (15)). The expression of the expected value of material possessions among representatives of various occupational groups was assumed as follows:

(21) $$P_{S}=P\left(X>-\varepsilon \right)$$

where X has the free tangent law $F(p_{n}^{e},\sigma_{n}^{2})$. This value estimates the probability of satisfaction from material possessions or the sum of responses of b and c. It was assumed that the lack of access to certain categories of material goods justified by the lack of need for such goods might safely be regarded as evidence of satisfaction from material possessions.

## 5. Practical Applications of the Postulated Model

The wealth of data collected from questionnaire surveys reflects preferences with regard to material possessions expressed by respondent households of the Lower Silesia region in 2021. The survey was done by means of structured questionnaires and face-to-face interviews (in-house survey). The dataset was segmented into three subsets: one for blue-collar workers employed in the construction trade (79 respondents), one for white-collar workers in the finance and administration sectors (81), and one for farmers (97). For all of the 4751 responses, 4626 = 18 × (79 + 81 + 97) valid questionnaires were obtained after screening the missing values for incomplete responses. Among them, there were 136 males and 121 females. The reliability assessment was evaluated according to the value of Cronbach’s alpha. Its value in this survey is 0.9012, exceeding the threshold of 0.7, which indicates the internal consistency and satisfies the reliability test.

Table 2 presents the structure of quantitative responses addressed to each of the three subsets collected with regard to each category of goods under study. Three variants of responses were available for each query, as described in Section 3. The last row presents values of the probabilities, $P_{S}$, for each subgroup calculated using Formula (21).

Values in the last row of Table 2 suggest a wide differentiation of expectations related to satisfaction from material possessions between the respondent subsets. Satisfaction from material possessions was the most pronounced among farmers, with construction workers placing second, and white-collar workers placing lowest on the scale. This may be relatable to the fact that blue-collar workers tend to place less emphasis on the quality of their material possessions and draw satisfaction from having access to such goods in the first place. One may speculate, at this point, that manual work may stimulate the feeling of contentment from each feat. After all, hard-earned resources are generally spent in more parsimonious ways. Representatives of this segment of the population display no need for replacing durable goods if they continue to serve their purpose. It may safely be observed that this segment of the general population is decidedly more effective in their exploitation of durable goods. This group is also more resilient to the widely emphasized marketing argument of novelty.

Compared to blue-collar staff, the employees of the financial and administrative sectors typically have the benefit of higher wages; this may arguably be the reason for their perceived emphasis on modern and state-of-the-art qualities of their material belongings. The pervading trend requires higher social status to be expressed in material ways. In effect, a car more than suitable for personal transportation in the eyes of a construction worker may not be satisfactory for a member of a financial profession, e.g., for technological reasons. The comparison of possession percentage of the distinctive group of luxury goods: own house, holiday house, and motorboat show that the order for each of the goods is the same:

Construction > Finance > Farming:

- Own house:66% > 30% > 7%,
- Holiday house:23% > 22% > 7%,
- Motorboat:30% > 22% > 3%.

On the other hand, the satisfaction from possessing goods is presented very differently:

Farming (Fr) > Construction (C) > Finance (Fn), more precisely: 0.7268 > 0.6358 > 0.5785.

The final order can be expressed by an odds ratio (OR), which can be calculated according to the formula:

$$OR_{A,B}=\frac{S\left(A\right)}{S\left(B\right)}=\frac{\frac{P\left(A\right)}{1-P\left(A\right)}}{\frac{P\left(B\right)}{1-P\left(B\right)}}=\frac{P\left(A\right)\cdot \left(1-P\left(B\right)\right)}{P\left(B\right)\cdot \left(1-P\left(A\right)\right)},$$

where *P*(*A*) is probability of an event in group A, and *S*(*A*) is the chance of the events’ occurrence.

The results are presented as follows, OR_Fr,C_ = 1,52; OR_Fr,Fn_ = 1,94; OR_C,Fn_ = 1,25. The comparison reveals that from the analysed groups, farming has the most appreciation for owning goods.

As evidenced above, the postulated model allows for the effective evaluation of differences in household satisfaction from material possessions between representatives of various social groups. The resulting evaluation is relative and offers the potential to place the results obtained from individual groups against the average values observed in other groups. As such, it may be adopted for ranking purposes. It must be noted that the model does not merely record the ownership/availability of any given category of goods but—above all—examines the satisfaction of household members produced by the availability of each item. For this reason alone, the postulated approach has considerable innovative value.

Future Directions: future studies should endeavour to focus on a range of different contexts and cohorts and include various types of measurement scales and response options. In particular, the dependence of satisfaction from possessing goods and general household welfare (e.g., expressed by the income of the man of the house) will be analyzed.

## Figures and Tables

**Table 1:** Possessions declared by respondent households (%).

Category of Goods	I Have Access I	I Don’t Own Any; I Have No Need for It II	I Don’t Own Any; ICannot Afford III	$p_{n}$
Automatic washing machine	93.83	2.54	3.63	92.74
LCD/plasma-screen TV	76.77	9.28	13.95	72.1
Internet access at home	66.52	28.04	5.44	89.12
Passenger car	64.37	23.39	12.24	75.52
Microwave oven	62.16	27.08	10.75	78.49
Portable computer	53.02	33.49	13.49	73.02
Own house	49.6	23.21	27.2	45.61
Paid satellite TV	44.48	43.18	12.34	75.32
Phone landline	43.31	48.8	7.89	84.22
Own flat	40.6	36.68	22.73	54.55
Desktop computer	39.41	52.51	8.08	83.84
Dish-washing machine	27.18	46.89	25.93	48.14
Ipador tablet	20.67	60.97	18.36	63.28
Allotment	11.63	58.98	29.38	52.51
Other type of real property	7.27	56.05	36.68	26.64
E-book reader	4.32	83.89	11.78	76.43
Holiday house	3.86	59.28	36.86	26.28
Motorboat	0.86	75.66	23.48	53.04

Source: own research, based on [22].

**Table 2:** Structure of survey responses.

	Construction			Finance			Farming		
Category of Goods	(a)	(b)	(c)	(a)	(b)	(c)	(a)	(b)	(c)
Automatic washing machine	17	6	56	27	6	48	21	4	72
LCD/plasma-screen TV	32	3	44	38	5	38	32	3	62
Internet access at home	4	10	65	36	2	43	41	11	45
Passenger car	32	3	44	46	2	33	53	6	38
Microwave oven	17	22	40	17	41	23	26	49	22
Portable computer	41	5	33	23	15	43	31	12	54
Own house	24	3	52	46	11	24	87	3	7
Paid satellite TV	3	27	49	31	13	37	20	33	44
Phone landline	19	46	14	9	68	4	9	39	49
Own flat	29	37	13	54	12	15	5	89	3
Desktop computer	16	35	28	29	8	44	21	26	50
Dish-washing machine	12	15	52	46	3	32	19	36	42
Ipad or tablet	21	31	27	24	12	45	14	56	27
Allotment	42	2	35	39	19	23	7	3	87
Other type of real property	57	5	17	47	23	11	12	2	83
E-book reader	38	5	36	27	45	9	28	59	10
Holiday house	51	10	18	41	22	18	52	38	7
Motorboat	49	6	24	51	12	18	22	72	3
$P_{S}$	0.6358			0.5785			0.7268		

Source: surveys and own studies.

## Data Availability

Available after asking the authors.
